# Genome mapping and characterization of the *Anopheles gambiae *heterochromatin

**DOI:** 10.1186/1471-2164-11-459

**Published:** 2010-08-04

**Authors:** Maria V Sharakhova, Phillip George, Irina V Brusentsova, Scotland C Leman, Jeffrey A Bailey, Christopher D Smith, Igor V Sharakhov

**Affiliations:** 1 Department of Entomology, Virginia Tech, Blacksburg, VA 24061, USA; 2 Department of Molecular and Cellular Biology, Institute of Chemical Biology and Fundamental Medicine, Siberian Branch of Russian Academy of Sciences, Novosibirsk 630090, Russia; 3 Department of Statistics, Virginia Tech, Blacksburg, VA 24061, USA; 4 Program in Bioinformatics and Integrative Biology and Department of Medicine, Division of Transfusion Medicine, University of Massachusetts Medical School, Worcester, MA 01605, USA; 5 Department of Biology, San Francisco State University, San Francisco, CA 94132, USA; 6 Drosophila Heterochromatin Genome Project, Lawrence Berkeley National Lab, Berkeley, CA 94720, USA

## Abstract

**Background:**

Heterochromatin plays an important role in chromosome function and gene regulation. Despite the availability of polytene chromosomes and genome sequence, the heterochromatin of the major malaria vector *Anopheles gambiae *has not been mapped and characterized.

**Results:**

To determine the extent of heterochromatin within the *An. gambiae *genome, genes were physically mapped to the euchromatin-heterochromatin transition zone of polytene chromosomes. The study found that a minimum of 232 genes reside in 16.6 Mb of mapped heterochromatin. Gene ontology analysis revealed that heterochromatin is enriched in genes with DNA-binding and regulatory activities. Immunostaining of the *An. gambiae *chromosomes with antibodies against *Drosophila melanogaster *heterochromatin protein 1 (HP1) and the nuclear envelope protein lamin Dm_0 _identified the major invariable sites of the proteins' localization in all regions of pericentric heterochromatin, diffuse intercalary heterochromatin, and euchromatic region 9C of the 2R arm, but not in the compact intercalary heterochromatin. To better understand the molecular differences among chromatin types, novel Bayesian statistical models were developed to analyze genome features. The study found that heterochromatin and euchromatin differ in gene density and the coverage of retroelements and segmental duplications. The pericentric heterochromatin had the highest coverage of retroelements and tandem repeats, while intercalary heterochromatin was enriched with segmental duplications. We also provide evidence that the diffuse intercalary heterochromatin has a higher coverage of DNA transposable elements, minisatellites, and satellites than does the compact intercalary heterochromatin. The investigation of 42-Mb assembly of unmapped genomic scaffolds showed that it has molecular characteristics similar to cytologically mapped heterochromatin.

**Conclusions:**

Our results demonstrate that *Anopheles *polytene chromosomes and whole-genome shotgun assembly render the mapping and characterization of a significant part of heterochromatic scaffolds a possibility. These results reveal the strong association between characteristics of the genome features and morphological types of chromatin. Initial analysis of the *An. gambiae *heterochromatin provides a framework for its functional characterization and comparative genomic analyses with other organisms.

## Background

Located in pericentric, telomeric, and some internal chromosomal regions, heterochromatin plays an important role in cell division [1], meiotic pairing [2], regulation of DNA replication, and gene expression [3]. Among insect species, the most detailed analysis of heterochromatin has been performed in *Drosophila *[4-7]. Molecular analysis has determined that pericentric heterochromatic regions are enriched with highly and moderately repetitive DNA sequences, and are extremely depleted of genes [8-10]. Mapping of heterochromatic scaffolds is difficult because the heterochromatin is underreplicated and poorly banded in polytene chromosomes of salivary glands. Special efforts had to be directed towards the assembly and annotation of heterochromatin in *Drosophila *[10-14]. Bioinformatic analysis of the heterochromatic portion of the *Drosophila *genome revealed the presence of more than 200 genes. Interestingly, heterochromatic genes are enriched specific functional domains, including putative membrane cation transporters domains and domains involved in DNA or protein binding [12]. This finding suggests that pericentric heterochromatin may encode genes involved in the establishment or maintenance of alternative chromatin states. In addition to the pericentric heterochromatin, *Drosophila *has intercalary heterochromatin, which is interspersed throughout the euchromatin and characterized, in part, by underreplication in polytene chromosomes of larval salivary glands [15,16]. A study of a genome-wide profile of underreplication in polytene chromosomes identified 52 underreplication zones, which were colocalized with regions of intercalary heterochromatin. These underreplication zones varied from 100 to 600 kb in length, and each contained from 6 to 41 unique genes [17].

One of the important problems of chromosome biology is to understand the relationships between the morphology of the chromatin and the DNA and protein composition. Two morphological types of the heterochromatin have been described in the pericentromeric regions of *Drosophila *polytene chromosomes: proximal condensed, α-, and distal diffuse, β-heterochromatin [18]. The compact central part of the chromocenter (α-type) is enriched with satellite DNA, while the distal diffuse area (β-type) contains mostly transposable elements (TEs) [19,20]. Biochemical studies have discovered that heterochromatic regions have a specific histone code, characterized by hypoacetylation and methylation of the histone H3 at lysine 9 [21]. This modification of the histone H3 is a docking site for the heterochromatin protein 1 (HP1) [22,23], a major component of heterochromatin first described in *Drosophila *[24]. Comparative studies of *Drosophila *polytene chromosomes have discovered differences in the chromatin state suggesting the switching of chromatin states during evolution. For instance, when staining patterns of HP1 on polytene chromosomes were compared, it was found that the heterochromatic fourth chromosomes of *D. melanogaster *and *D. pseudoobscura *bind to HP1, while the euchromatic fourth chromosome of *D. virilis *does not. Interestingly, the level of CA/GT repeats on chromosome 4 of *D. virilis *is 20 fold higher than the level on chromosome 4 of *D. melanogaster*. Moreover, the density of TEs in this chromosome is significantly higher for *D. melanogaster *than for *D. virilis *[25-27].

A number of studies have demonstrated direct associations between heterochromatin and the nuclear envelope (NE) [28-33]. In *Drosophila *salivary gland nuclei, pericentromeric heterochromatin attaches permanently to the NE, while intercalary heterochromatin forms high-frequency contacts to NE [34]. Chromatin fibers of diffuse heterochromatin form visible attachments to the NE in *Drosophila *[30] and *Anopheles *[35,36]. The chromosomal regions that attach to the NE may depend on the presence of specific DNA. For example, repetitive matrix attachment regions (MARs) specifically bind to lamin, the major protein of the nuclear periphery [28,37-40]. It has been shown that MAR DNA is several fold richer in heterochromatin than in euchromatin [41-43].

Although the *Drosophila *studies provided important insights into the structural and functional organization of heterochromatin, the organization of heterochromatin in other insects remains poorly understood. Malaria mosquitoes are an excellent system for studying heterochromatin because they possess well-developed polytene chromosomes with clear morphology. Sequencing of the genome of the major African malaria vector *An. gambiae *[44] provides an opportunity to analyze the molecular structure of the heterochromatin and to study genomic determinants of heterochromatin formation, maintenance, and function. In malaria mosquitoes, the heterochromatin size and morphology vary significantly among species and within species [45-47], affecting mating behavior and fertility [48,49]. In the *An. gambiae *complex, one of the species, *An. gambiae sensu stricto*, is subdivided into two subtaxa: the M and S molecular forms [50]. These two partially isolated subtaxa predominantly breed within their own form and differ in behavior and environmental adaptations [51]. A DNA microarray analysis revealed that two pericentric regions on X and 2L were the major islands of fixed genomic differentiation between the M and S molecular forms [52]. A more recent microarray study based on the improved AgamP3 assembly and AgamP3.4 gene build provided better estimates for the number and size of diverged pericentric islands between the M and S forms [53]. The study found three islands of genomic divergence: a ~4-Mb region on the X chromosome, a ~2.5-Mb region on the 2L arm, and a 1.7-Mb region on the 3L arm. However, it is not clear if the pericentric islands of genomic divergence are located within heterochromatin or mostly overlap with euchromatin of *An. gambiae.*

According to the C_o_T analysis, about 86 Mb (33% of 260-Mb genome) of the *An. gambiae *genome corresponds to repetitive elements, which are mostly located in heterochromatic areas of the chromosomes [54]. However, only 3.3 Mb were identified as heterochromatin in the first publication of *An. gambiae *genome [44]. Using cDNA clones for the physical mapping of the heterochromatic scaffolds, an additional 5.3 Mb were mapped to the pericentromeric regions in the chromosomes [55]. Nevertheless, the more precise chromosomal and genomic mapping, as well as detailed analysis of the molecular organization of the *Anopheles *heterochromatin, has yet to be conducted.

In this study, the boundaries of the heterochromatin-euchromatin junctions of all morphologically defined pericentric and intercalary heterochromatin regions were determined for each of the five chromosomal arms of *An. gambiae*. The large regions of intercalary heterochromatin were morphologically different: 0.7-Mb and 0.8-Mb regions of 2L and 3L were diffuse, while a 2.9-Mb region of 3R was a compact heterochromatin. Because the *An. gambiae *genome assembly successfully captured not only the euchromatin, but a significant portion of the heterochromatin, comparative analysis of chromatin types was possible. We provided evidence that heterochromatin and euchromatin differ in gene density and the coverage of retroelements and segmental duplications (SDs). Gene ontology (GO) analysis revealed that heterochromatin is enriched in genes with DNA-binding and regulatory activities. The pericentric heterochromatin had the highest coverage of retroelements and tandem repeats, while intercalary heterochromatin was enriched with SDs. We also demonstrated that the diffuse intercalary heterochromatin binds to HP1 and lamin and has a higher coverage of DNA TEs, minisatellites, and satellites than does the compact intercalary heterochromatin. The investigation of 42-Mb assembly of unmapped genomic scaffolds ("unknown chromosome") demonstrated that it has molecular characteristics similar to cytologically mapped heterochromatin. Finally, the locations and sizes of pericentric heterochromatin regions closely matched the locations and sizes of pericentric islands of genomic divergence between M and S incipient species of *An. gambiae*.

## Results and Discussion

### Morphological types of the *An. gambiae *heterochromatin

**Figure 1:**
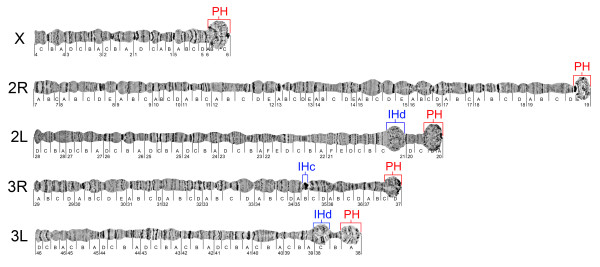
**The pericentric and intercalary heterochromatin of polytene chromosomes shown on a standard cytogenetic map of *An. gambiae ***[91]. PH--pericentric heterochromatin, IHc--compact intercalary heterochromatin, IHd--diffuse intercalary heterochromatin.

**Figure 2:**
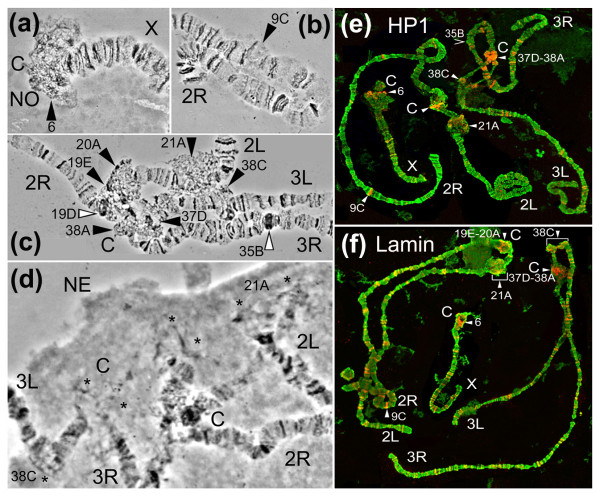
**Localization of HP1 and lamin Dm_0 _*Drosophila *antibodies on *An. gambiae *chromosomes**. Small numbers and letters indicate subdivisions of the chromosome map. The diffuse type of heterochromatin is shown by black arrowheads (a, b, c). The white arrowheads show compact heterochromatin (c) and sites of HP1 and lamin localization (e, f). Asterisks (d) show attachments of diffuse heterochromatin to the NE. X, 2R, 2L, 3R, 3L - chromosomal arms, C - centromeric areas.

The diploid number of the chromosomes in malaria mosquitoes is six, which includes two pairs of autosomes as well as the X and Y sex chromosomes. The polytene chromosome complement of a female mosquito has five chromosomal arms: four autosomal arms 2R, 2L, 3R, 3L, and one arm of the X chromosome. In this study, morphological identification of the heterochromatin for the African malaria mosquito *An. gambiae *was performed for the first time. The following criteria were used to distinguish heterochromatic and euchromatic regions in the polytene chromosomes from ovarian nurse cells (Figure 1). We considered a region as heterochromatic if it (i) consisted of a compact condensed block or (ii) had a diffuse granulated structure with no banding pattern. These two types of heterochromatin can be distinguished from euchromatic regions, which have a clear banding pattern or puffy nongranulated areas. Pericentric regions of all chromosomes matched these morphological criteria of heterochromatin. The pericentric heterochromatin of the X chromosome has a large diffuse granulated area in region 6, which is similar to the β-heterochromatin of *Drosophila *(Figure 2a). The diffuse granulated heterochromatin (Figure 2a) is morphologically distinct from the euchromatic nongranulated puff in subdivision 9C of the 2R arm (Figure 2b). In addition, region 6 of the X chromosome has a dark compact band in the tip of the chromosome (Figure 1), which was previously described as a nucleolar organizer region because ribosomal genes were mapped to this area by *in situ *hybridization [55]. The polytene chromosome 2 has a dark compact proximal heterochromatin surrounded by abundant diffuse heterochromatin in regions 19E-20A (Figure 2c). A dark heterochromatic band is also present in region 19D of the 2R arm. The pericentric heterochromatin of chromosome 3 spans subdivisions 37D-38A. Chromosomes 2 and 3 form a diffuse chromocenter via their pericentric heterochromatin [36].

Three regions of intercalary heterochromatin are visible on arms 2L, 3R, and 3L (Figure 2c). The subdivision 21A of 2L chromosomal arm forms a large, lightly granulated puff-like structure with no banding pattern. The middle area of subdivision 38C of 3L arm has a similar morphology, but it is slightly smaller and darker. Both regions of intercalary diffuse heterochromatin are located in close proximity to the pericentric regions. The third region of intercalary heterochromatin is in subdivision 35B of the 3R arm and is located 10 subdivisions away from the centromere. Unlike intercalary heterochromatin of 2L and 3L, this region has a compact dense structure, which is similar to α-heterochromatin of *Drosophila. *In malaria mosquitoes, diffuse and compact types of heterochromatin were previously described in the *Anopheles maculipennis *subgroup [35,56]. Interestingly, the large blocks of compact heterochromatin or the diffuse intercalary heterochromatin regions have not been seen in most species of *Drosophila. *The intercalary heterochromatin in salivary gland nuclei of *D. melanogaster *is strongly underreplicated and has the morphology of ''weak'' points, which are able to form ectopic contacts [57]. These properties are less prominent in ovarian nurse cell nuclei of the *D. melanogaster otu*^*11 *^strain where the bands of intercalary heterochromatin are morphologically similar to euchromatic bands [58]. Large blocks of intercalary heterochromatin have been described in polytene chromosomes of *D. immertensis *and species from genera *Chironomus *and *Anopheles *[4,56]. Although the morphology of pericentric heterochromatin is similar in *An. gambiae *and *D. melanogaster*, the presence of two distinct types of intercalary heterchromatin in *An. gambiae *makes this species a unique model system for studying genomic determinants of chromatin morphology.

### Chromosomal localization of HP1 and lamin in *An. gambiae*

HP1 is an evolutionarily conserved protein and a good marker of heterochromatic regions [25]. One *HP1a *ortholog is present in *An. gambiae *(VectorBase gene ID: *AGAP009444*). The *An. gambiae *protein AGAP009444-PA is 70.4% similar to the *D. melanogaster *HP1a protein in the 206 overlapping amino acids. The antibodies for HP1 were localized in the chromocenter, chromosome 4, telomeric, and some euchromatic regions in *D. melanogaster *[24,59]. In order to examine the association of HP1 with heterochromatin in *An. gambiae*, we hybridized the primary antibody C1A9 against *D. melanogaster *HP1 to *An. gambiae *polytene chromosomes. This antibody correctly recognized HP1 even in more distantly related species such as the mealybug *Planococcus citri *[60]. Several positively stained loci were invariable, i.e., they were found on every examined chromosome and on every slide. Similar to *Drosophila*, the major invariable sites of HP1 localization were the pericentric regions in *An. gambiae *(Figure 2). In addition, diffuse intercalary heterochromatin of regions 21A and 38C were always stained positively for HP1. Only one major invariable HP1-binding site was identified in a large interband of the euchromatic region 9C of 2R arm (Figure 2b). All other positive euchromatic sites were variable, and a total of 122 HP1 binding sites were detected on *An. gambiae *chromosomes (Table 1). Based on the previous *An. gambiae *genome mapping coordinates, we analyzed the molecular content of the euchromatic site of HP1/lamin binding in region 9C (genome coordinates 12874430-13778780). The analysis found no enrichment of any class of TE. The only heterochromatic molecular feature of this region was a 4.5-kb block of satellite DNA, which consisted of 228-bp units repeated 40 times. Similarly, one major invariable site of HP1 binding was found in euchromatic region 31 of the 2L arm in *D. melanogaster *[61]. However, the molecular analysis of this region found no enrichment in any repetitive DNA. About 200-300 actively expressing loci related to developmentally important and heat-shock genes were positively stained for HP1 in *Drosophila *chromosomes, suggesting a positive role for HP1 in euchromatic gene expression [62-64]. However, only 20 HP1-positive euchromatic sites were invariable among strains, natural populations, and individuals of *D. melanogaster *[61]. Unlike in *Drosophila*, telomeric localization of HP1 was found only on chromosome X in *An. gambiae*, but even this site was variable. Surprisingly, no HP1 binding was detected in the compact intercalary heterochromatin of subdivision 35B of the 3R chromosome, suggesting that this region has a distinct molecular composition or is strongly underreplicated, and thus, HP1 presence is below the level of detection. Subdivision 35B was morphologically described as heterochromatic based on very dense dark structure (Figure 1 and 2c). The genomic analysis confirmed its repeat-rich gene-poor heterochromatic nature (see "Difference in molecular content among chromatin types of *An. gambiae*").

**Table 1:** Localization of HP1 and lamin on *An. gambiae *chromosomes

Chromosome	Invariable sites	Variable sites	Average number of variable sites per Mb	Chromosome analyzed
X: HP1	1	1-17	0.33	10
X: Lamin	1	1-19	0.41	5

2R: HP1	2	13-53	0.55	3
2R: Lamin	2	7-49	0.46	5

2L: HP1	2	2-20	0.22	4
2L: Lamin	2	17-31	0.49	5

3R: HP1	1	1-18	0.18	3
3R: Lamin	1	29	0.55	2

3L: HP1	2	2-14	0.19	2
3L: Lamin	2	30	0.71	1

Association of heterochromatin with the NE has been demonstrated in a number of studies [28-33]. Attachment of pericentric regions to the NE in ovarian nurse cell nuclei of *An. gambiae *has also been demonstrated [36]. In our study, the attachments to the nuclear periphery were detected in all pericentric regions, and diffuse intercalary heterochromatin in regions 21A (2L) and 38C (3L) (Figure 2d). To test whether heterochromatin binds to the NE, mosquito chromosomes were stained with antibody ADL67.10 against NE protein lamin Dm_0 _of *D. melanogaster*. We found only one *lamin Dm0 *ortholog in the *An. gambiae *genome (VectorBase gene ID: *AGAP011938*). The *An. gambiae *protein AGAP011938-PA is 78.2% similar to the *D. melanogaster *lamin Dm_0 _protein in the 628 overlapping amino acids. The antibody against lamin Dm_0 _successfully hybridized to the *An. gambiae *chromosomes and colocalized with the HP1 antibody in all major invariable sites and in most of the variable sites (Figure 2f). However, the total number of sites was higher for lamin Dm_0 _(158 sites) than for HP1 (122 sites) (Table 1). The major sites for lamin Dm_0 _were found in the pericentromeric areas, diffuse intercalary heterochromatin regions, and euchromatic interband in region 9C. No lamin Dm_0 _antibody was detected in region 35B of the 3R chromosome of *An. gambiae.*

Thus, the immunostaining of the antibodies for HP1 and lamin Dm_0 _has demonstrated that both proteins are primarily associated with the diffuse pericentric and intercalary heterochromatin, but not with the compact intercalary heterochromatin of *An. gambiae. *Two binding motifs, chromo and chromoshadow domains, provide HP1 with the ability to be broadly involved in chromatin and protein binding [65-67]. *In vitro *studies revealed a direct interaction between HP1 and the lamin B receptor in mammalian cells [33,68,69]. However, in *Drosophila*, similar direct associations of HP1 with lamin have not been shown, and these proteins have been found associated with different genomic regions [70]. Therefore, despite the colocalization of HP1 and lamin in heterochromatin of *An. gambiae*, the actual protein binding sites in the genome may differ as suggested by the additional regions of lamin binding.

### Heterochromatin-euchromatin boundaries in the *An. gambiae *genome

**Figure 3:**
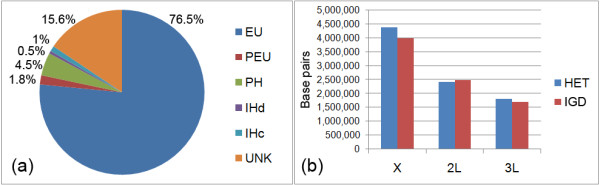
**Schematic representation of the heterochromatin amount in the *An. gambiae *genome**. (a) Relative proportions of mapped chromatin types and unmapped sequences in the assembly. PH--pericentric heterochromatin, IHc--compact intercalary heterochromatin, IHd--diffuse intercalary heterochromatin, PEU--proximal euchromatin, EU--euchromatin, UNK--"unknown chromosome." (b) Comparison of sizes and positions of islands of genomic divergence (IGD) and regions of pericentric heterochromatin (HET) in the X chromosome, the 2L arm, and 3L arm. Position of a putative centromere corresponds to 0 bp.

The cytological identification of heterochromatin allowed us to determine the location of heterochromatin-euchromatin boundaries in the *An. gambiae *genome. The approximate coordinates were found based on the genome positions of BAC and cDNA clones, which were physically mapped to chromosomes near heterochromatin-euchromatin boundaries [44,55]. Because heterochromatic regions were not sufficiently covered with markers, additional PCR-amplified gene fragments were designed and utilized as DNA probes for physical mapping. Fluorescent *in situ *hybridization (FISH) was used to hybridize multiple PCR products thought to be located near the heterochromatin-euchromatin boundary of each major heterochromatic region of the five chromosome arms (Table 2). This allowed for more exacting definition of the boundaries, based on the outermost heterochromatin and euchromatin markers, defining a transition zone with an average size of 78 kb (range: ~15 to 226 kb). Based on these boundaries, a total of ~16.6 Mb was defined as a heterochromatin in the currently mapped genome assembly of *An. gambiae *(Figure 3a). The mapped portion of the heterochromatin within defined chromosomes now comprises ~6.4% of the ~260-Mb genome [44,54] and contains 232 (~1.8%) of the ~13,000 total predicted genes. For comparison, no less than 230 genes were annotated in 24 Mb of *D. melanogaster *heterochromatin (release 5.1) [12]. In addition, the sizes of intercalary heterochromatin were also determined. The diffuse heterochromatic regions were 0.7 Mb and 0.8 Mb in 2L and 3L, respectively, and the compact heterochromatin on 3R was 2.9 Mb long. The relatively short sizes of regions of intercalary diffuse heterochromatin as compared to regions of condensed heterochromatin suggest incomplete genome assembly of the diffuse type. However, these sizes exceed the sizes of intercalary heterochromatin known in *Drosophila*, which range from 100 to 600 kb [17]. The higher repeat content of the mosquito genome may be responsible for the larger sizes of intercalary heterochromatin in *An. gambiae.*

**Table 2:** Boundaries between heterochromatin and euchromatin in the *An. gambiae *genome.

Chromosome		Genome coordinates		Mapped markers			
	**Chromatin type**	**Start**	**End**	**Start**	**End**	**Size (bp)**	**Gene number**

**X**							

	EU	1	19,928,574	Telomere	AGAP001035	19,928,573	1035

	CH	20,009,764	24,393,108	AGAP001039	Centromere	4,383,344	56

**2R**							

	EU	1	58,969,802	Telomere	AGAP004644	58,969,801	3550

	CH	58,984,778	61,545,105	26D02	Centromere	2,560,327	32

**2L**							

	CH	1	2,431,617	Centromere	AGAP004707	2,431,616	31

	PEU	2,487,770	5,042,389	AGAP004711	AGAP004892	2,554,619	183

	IH	5,078,962	5,788,875	AAAB01008948_1	AGAP004905	709,913	12

	EU	6,015,228	49,364,325	AGAP004919	Telomere	43,349,097	2812

**3R**							

	EU	1	38,815,826	Telomere	AGAP009690	38,815,825	1960

	IH	38,988,757	41,860,198	AGAP009696	AGAP009730	2,871,441	35

	EU	41,888,356	52,131,026	BAC 30P16	BAC 25H11	10,242,670	554

	CH	52,161,877	53,200,684	AGAP010287	Centromere	1,038,807	23

**3L**							

	CH	1	1,815,119	Centromere	AGAP010342	1,815,118	33

	PEU	1,896,830	4,235,209	AGAP010344	AGAP010481	2,338,379	138

	IH	4,264,713	5,031,692	AGAP010482	AGAP010491	766,979	10

	EU	5,133,257	41,963,435	AGAP010505	Telomere	36,830,178	1923

### Heterochromatin and pericentric regions of genomic divergence in incipient species

Three pericentric islands of genomic divergence were found in chromosomes X, 2L, and 3L in two partially isolated subtaxa - the M and S molecular forms of *An. gambiae *s.s. [53]. Our analysis showed that the positions of islands of genomic divergence mostly correspond to the positions of physically mapped regions of pericentric heterochromatin (Figure 3b). The sizes of the pericentric heterochromatin were the following: 4.4 Mb of the X chromosome, 2.4 Mb of the 2L arm, and 1.8 Mb of the 3L arm. Thus, the overlaps with islands of genomic divergence are 91% in the X chromosome, 97% in the 2L arm, and 94% in the 3L arm. This observation suggests that heterochromatic sequences diverge rapidly during speciation of malaria mosquitoes. Earlier cytological studies showed the presence of significant intra- and interspecific differences in amount and location of heterochromatin in the *An. gambiae *complex [45,48]. A genome-wide microsatellite study of members of the *An. gambiae *complex has determined a high level of genetic introgression among species [71]. However, the *An. gambiae *microsatellites at six loci of X, 3L, and 3R could not be amplified in all sibling species, indicating significant sequence divergence from the major malaria vector. These loci were identified as heterochromatic in our study. Fast changes in heterochromatic DNA can be accompanied by the rapid evolution of heterochromatic proteins. Although HP1 is an evolutionarily conserved protein, other heterochromatin- and centromere-associated proteins demonstrate rapid adaptive evolution [72,73]. For example, an LHR protein encoded by *lhr *(*Lethal hybrid rescue) *colocalizes with HP1 in heterochromatic regions and has diverged extensively in sequence between *D. melanogaster *and *D. simulans *species in a manner consistent with positive selection. Interestingly, F1 hybrids between these species demonstrate altered chromatin structure, probably attributable to the effects of species-specific differences in TEs and other repetitive DNAs [74], suggesting a role for heterochromatin in speciation.

### Overrepresentation of gene ontology terms in the *An. gambiae *heterochromatin

**Figure 4:**
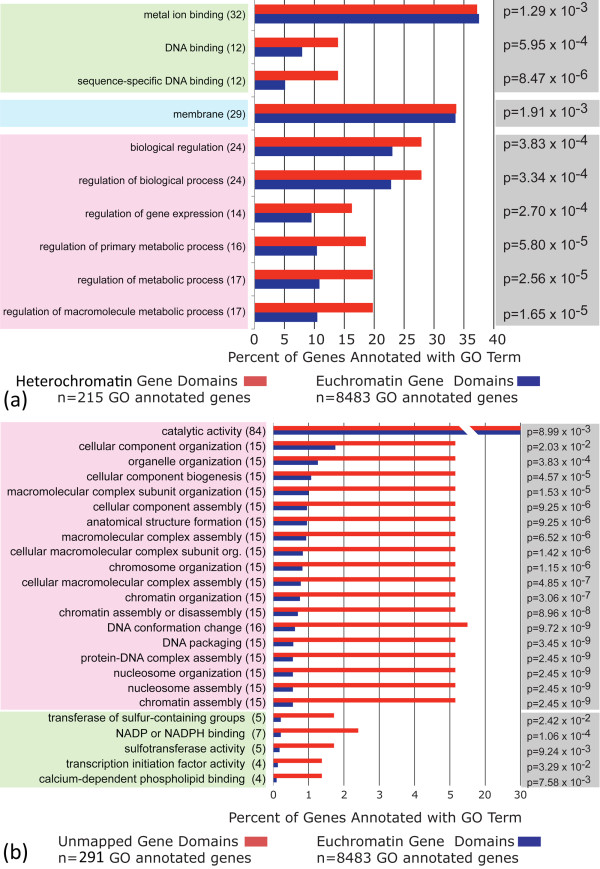
**Overrepresented GO terms in genes within the cytologically confirmed heterochromatin (a) and within "unknown chromosome" (b) of *An. gambiae***. The percentages of heterochromatic (red) and euchromatic (blue) genes containing the listed GO biological process (pink shading), cellular location (blue shading), and molecular function (green shading) terms are indicated. Numbers in parentheses refer to the actual number of heterochromatin or unmapped genes annotated with the listed GO domain. GO-Term-Finder, Bonferroni corrected p-value scores are shown to the right (grey shading).

To characterize gene content of the *An. gambiae *heterochromatin, we utilized GO terms [75]. The frequencies of GO terms assigned to genes in heterochromatin were compared to frequencies for all GO-annotated genes in the peptide dataset of *An. gambiae *(Figure 4a). After Bonferroni correction for multiple tests, this analysis revealed significant enrichment for molecular functions in heterochromatin, including DNA binding (12 genes) and sequence-specific DNA binding (12 genes). Protein products of 29 heterochromatic genes constitute membrane, representing a significant enrichment of the GO cellular location. Finally, heterochromatin had overrepresentation of several gene types, including those encoding for proteins involved in biological regulation (24 genes) and regulation of metabolic processes (17 genes) (biological processes). The GO analysis of the "unknown chromosome" (sequence assembly lacking chromosomal assignment) identified enrichment in a number of interesting genes (Figure 4b). We found that genes residing in the "unknown chromosome" had significant overrepresentation of GO terms in biological processes, including chromosome organization (15 genes), DNA packaging (15 genes), and nucleosome assembly (15 genes). Transcription initiation factor activity (four genes) was among several molecular functions overrepresented in the genes within the "unknown chromosome." Analysis of the heterochromatic portion of the *Drosophila *genome revealed the overrepresentation of similar GO terms [12]. These studies suggest that heterochromatin of insects may accumulate genes important for its own establishing, maintaining, or modifying chromatin structure.

### Difference in molecular content among chromatin types of *An. gambiae*

Using Bayesian statistical model and procedure for discerning differences between chromatin types, eight molecular features were analyzed: genes, DNA-mediated TEs (DNA TEs), RNA-mediated TEs (RNA TEs), SDs, micro- and minisatellites, satellites, and MARs. These molecular features were compared among five distinct chromatin types: 1) pericentric heterochromatin of all chromosomes; 2) diffuse intercalary heterochromatin in regions 21A of 2L and 38C of 3L; 3) compact intercalary heterochromatin, region 35B of 3R; 4) proximal euchromatin, located between pericentric and diffuse intercalary heterochromatin, includes subdivisions 20CD of 2L and 38B of 3L; and 5) euchromatin in all remaining regions in the chromosomes. For this analysis, the data that distinguishes both the counts and the overall base-pair coverage were incorporated for each molecular feature into the genomic windows of each of the five chromatin types. Dominant model selection procedures gave us the ability to compare all possible competing models and to select between parsimonious models by maximizing the posterior distribution.

**Figure 5:**
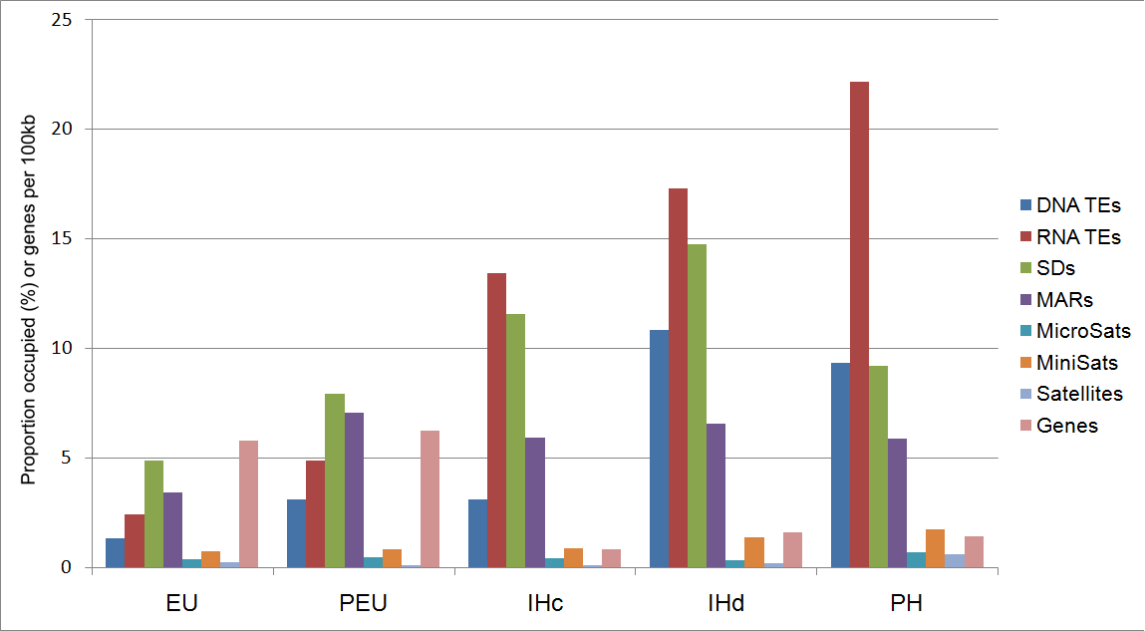
**Median values of gene density and repetitive element coverage in chromatin types of *An. gambiae***. Percentage of region length occupied per 1 Mb are indicated for all repetitive elements. PH--pericentric heterochromatin, IHc--compact intercalary heterochromatin, IHd--diffuse intercalary heterochromatin, PEU--proximal euchromatin, EU--euchromatin.

Heterochromatin had a uniformly low concentration of genes. On average, the gene density was 4.7 times lower in the heterochromatin than in the euchromatin (Additional file 1, Table S1). Our analysis showed that heterochromatin significantly exceeds euchromatin in the coverage of RNA TEs and SDs. RNA TEs were the most abundant features in the mosquito genome (Figure 5). The pericentric heterochromatin had the highest coverage of RNA TEs, microsatellites, minisatellites, and satellites. The intercalary heterochromatin had a higher coverage of SDs than all other chromatin types. The diffuse intercalary heterochromatin had a higher coverage of TEs, minisatellites, and satellites than did the compact intercalary heterochromatin. The enrichment of TEs in the pericentric heterochromatin and diffuse intercalary heterochromatin as compared to the compact intercalary heterochromatin can explain the pattern of HP1 localization in polytene chromosomes of *An. gambiae*. Pericentric and diffuse intercalary heterochromatin, but not the compact type, was HP1 positive. Similarly, the fourth chromosomes of *D. melanogaster *and *D. pseudoobscura *bound to HP1, while the fourth chromosome of *D. virilis *did not. The density of TEs in this chromosome was significantly higher for *D. melanogaster *than for *D. virilis *[25-27]. The proximal euchromatin had a higher coverage of DNA TEs, MARs, and SDs but a lower coverage of satellites than the rest of the euchromatin. These differences can probably be explained by the close distance of the proximal euchromatin to the centromere.

### Chromatin types and genome landscape in *An. gambiae*

**Figure 6:**
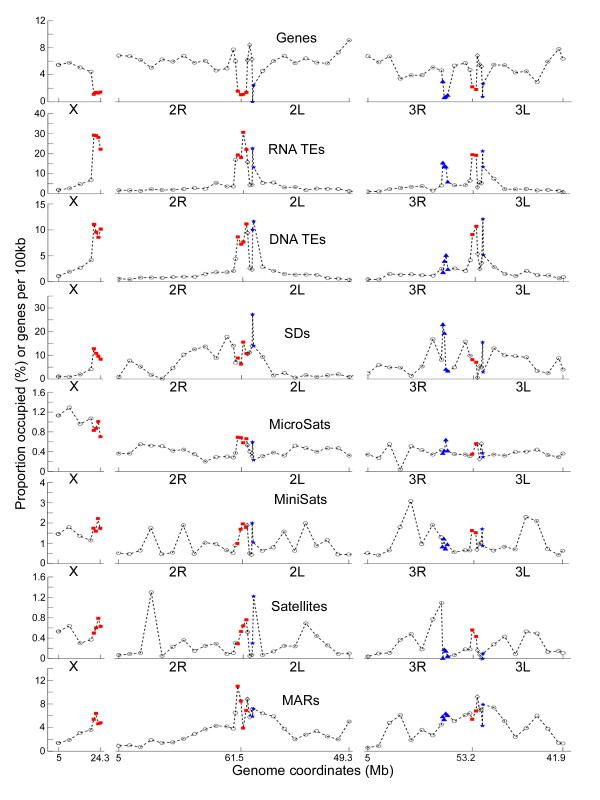
**Genome landscapes of the *An. gambiae *heterochromatin and euchromatin**. Median values of coverage of molecular features are displayed as 5-Mb intervals in euchromatin (open circles) and < 1-Mb intervals in heterochromatin. Red squares--pericentric heterochromatin, open diamonds--proximal euchromatin, blue stars--diffuse intercalary heterochromatin, blue triangles--compact intercalary heterochromatin.

In addition to the overall differences among chromatin types, the distribution of molecular features within chromosomal arms was analyzed. A high density of genes was seen outside of the heterochromatin boundaries believed to be euchromatin, followed by a transition zone and a heterochromatic region with a low gene density. The distribution of TEs densities had the opposite pattern. The highest coverage of SDs was detected in intercalary heterochromatin with peaks in some euchromatic regions of the 2R, 3R, and 3L arms (Figure 6). MARs were found concentrated in the pericentric heterochromatic and proximal euchromatic regions of all arms, but they were also abundant in distal euchromatic regions of the 2L, 3R, and 3L arms. We observed the high coverage of predicted MARs in heterochromatic regions, which are associated with the NE [41-43]. Moreover, the increase in MAR coverage seen in euchromatic regions of the 2L, 3R, and 3L arms correlated positively with the higher density of lamin-positive sites in these arms detected by immunostaining (Table 1). The highest coverage of MARs was found in proximal euchromatin, which was not stained by the lamin antibody. Also, the two types of heterochromatin were not significantly different in MAR coverage. However, the lamin-positive pericentric and diffuse intercalary heterochromatic regions were significantly enriched with TEs. The coverage of DNA TEs was about two times higher in pericentric and diffuse intercalary heterochromatin than in other chromatin types (Table S1).

Overall, this analysis confirmed morphological predictions of heterochromatin. All types of heterochromatin in the *An. gambiae *genome had typical heterochromatic molecular features: low gene density and high coverage of TEs and SDs. However, because TEs are significantly underannotated in the *An. gambiae *genome, meaningful comparisons of the TE content of heterochromatin between mosquito and fruit fly are difficult.

### Unmapped genome assembly of *An. gambiae*

**Figure 7:**
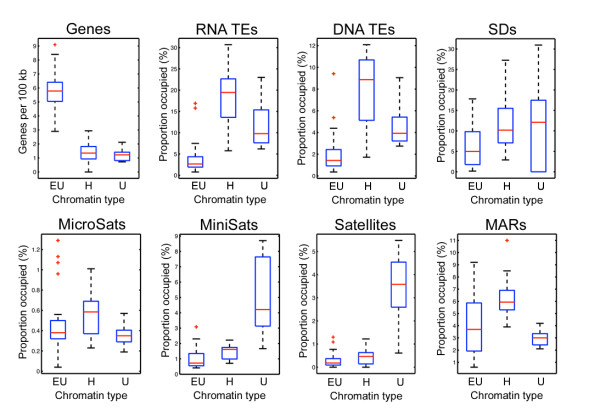
**Median values of gene density and repetitive element coverage in "unknown chromosome" of *An. gambiae***. EU--total euchromatin, H--total heterochromatin, U--"unknown chromosome."

The unmapped portion of the AgamP3 *An. gambiae *genome assembly comprises 42 Mb [76], i.e.~16% of the genome, and has 491 protein coding genes (Figure 3a). The analysis of the genomic content of this "unknown chromosome" (http://www.vectorbase.org/) revealed that the density of genes and the coverage of TEs and microsatellites were similar to that of the heterochromatin (Figure 7). The highest coverage of minisatellites and satellites was detected in the "unknown chromosome" suggesting that the majority of these scaffolds belong to heterochromatin. Two satellites, AgY477 and Ag53C, were mapped to the most proximal heterochromatin of the *An. gambiae *polytene chromosomes [77]. The location of satellite DNA in the proximal pericentric heterochromatin has also been demonstrated in *An. stephensi *[78]. An enrichment with highly repetitive DNA has been found in the compact heterochromatin of the *An. macullipennis *subgroup [56]. Telomeres of the *An. gambiae *chromosomes do not display heterochromatic morphology. Subtelomeric regions possess typical euchromatic banding patterns. However, molecular analysis of the telomeric end of the 2L arm demonstrated the presence of satellites and minisatellites [79,80]. Therefore, the unmapped portion of the *An. gambiae *genome assembly likely contains sequences from the most proximal pericentric, most distal telomeric ends of chromosomes, and intercalary diffuse heterochromatin. In *D. melanogaster*, 10 Mb of the unmapped portion of the genome was also enriched in tandem repeats and satellites [12].

## Conclusion

Morphological identification and detailed physical mapping allowed us to define an expanded compartment of recognizable heterochromatin with distinct molecular features within the *An. gambiae *genome assembly. Now about 16.6 Mb of mapped heterochromatin with 232 protein-coding genes is available for further characterization. GO analysis revealed that heterochromatin is enriched in genes that encode for proteins that may be involved in epigenetic regulation of chromatin. This study described the large regions of intercalary heterochromatin with a morphology not seen in *D. melanogaster*. We also provided evidence that heterochromatin and euchromatin significantly differ in gene density and the coverage of RNA TEs and SDs. The sequence composition, in terms of DNA TEs, RNA TEs, minisatellites, and satellites, can differentiate between the diffuse and compact types of intercalary heterochromatin. Conversely, MARs are distributed regardless of the chromatin type. The results of immunostaining with HP1 and lamin confirmed the general principle of nuclear organization--that the gene-poor regions of the genome reside at the nuclear periphery. Future investigations of *An. gambiae *heterochromatin need to show whether specific molecular composition can actually lead to chromosome-NE interactions. Given that the 42-Mb-long "unknown chromosome" has the molecular characteristics of heterochromatin, it is possible that only one third of heterochromatic sequences in the *An. gambiae *genome assembly have been placed to chromosomes. Finally, we found that pericentric islands of genomic divergence between M and S incipient species of *An. gambiae *are almost completely heterochromatic, demonstrating the elevated evolutionary plasticity of the mosquito heterochromatin.

## Methods

### Mosquito strain and chromosome preparation

A laboratory SUA strain of *An. gambiae *was used in this study. Mosquitoes were reared at 28°C at 80% humidity. Mosquitoes were grown at a low density (500-750 mosquitoes per 4 liter pan) to obtain better quality chromosomes. Larvae were fed *ad libitum*. Adults were given sugar water through dampened cotton balls that were removed at least 2 hours preblood feeding to ensure that most mosquitoes would take a blood meal. To obtain the chromosomal preparations, females were blood fed twice with a Guinea pig. Chromosomal slides for the morphological analysis were prepared as described previously [81]. Images were recorded with an Olympus Q-color5 digital cooled 5 megapixel camera and the Olympus CX41 light microscope using 1000× magnification (Olympus America Inc., Melville, NY, USA).

### Probe preparation and FISH

Genomic DNA from *An. gambiae *mosquitoes was isolated via a DNeasy Blood and Tissue Kit (Qiagen Inc., Valencia, CA, USA). PCR probes were chosen from the euchromatin--heterochromatin transition zones of the *An. gambiae *genome. Many of these probes were based on genes located near expected heterochromatin-euchromatin boundaries on each chromosome arm. Primers were designed using the Primer3 program [82]. PCR products ranged from 400-600 bp in size. The *in situ *hybridization procedure was done as previously described [81]. PCR products were gel purified using the Geneclean kit (Qbiogene, Inc., Irvine, CA). The DNA was labeled with Cy3-AP3-dUTP (GE Healthcare UK Ltd., Buckinghamshire, England) using the Random Primer DNA Labeling System (Invitrogen Corporation, Carlsbad, CA, USA). DNA probes were hybridized to the chromosomes at 39°C overnight in hybridization solution (Invitrogen Corporation, Carlsbad, CA, USA). Then the chromosomes were washed in 0.2 × SSC, (Saline-Sodium Citrate: 0.03 M Sodium Chloride, 0.003 M Sodium Citrate) counterstained with YOYO-1, and mounted in DABCO. Fluorescent signals were detected and recorded using a Zeiss LSM 510 Laser Scanning Microscope (Carl Zeiss MicroImaging Inc., Thornwood, NY, USA).

### HP1 and lamin antibodies immunolocalization

The original method of chromosome immunostaining was slightly modified for application to ovarian nurse cell polytene chromosomes [64,83]. In order to obtain polytene chromosomes from ovarian nurse cells, we blood fed female mosquitoes and kept them at regular conditions (temperature 26°C, humidity 80%) over night for 25 hours. Then half gravid females were placed on ice, and their ovaries were dissected. Every ovary was divided into two parts; each part was placed in fixative solution (47% water, 45% acetic acid, and 8% formaldehyde) separately; and follicles were spread on the slide by needles. Afterwards, the fixative solution was removed by filter paper, and follicles were placed in a fresh drop of the solution. Follicles were squashed under a cover slip and frozen in liquid nitrogen. Then cover slips were removed, and slides were kept in 70% cold ethanol at -20°C for several hours. Just before immunohybridization, slides were washed in PBS saline buffer (Boston Bioproduct, Worcester, MA, USA) with 0.1% Nonidet P40 and incubated for 20 minutes in blocking solution (1% BSA in PBS).

Primary mouse monoclonal antibodies C1A9 for Heterochromatin Protein 1 of *D. melanogaster *and ADL67.10 for *Drosophila *lamin Dm_0 _(Developmental Studies Hybridoma Bank, The University of Iowa, USA) were used for immunostaining of *An. gambiae *polytene chromosomes. Primary antibodies were diluted in 1:50 ratio and incubated overnight with the chromosomes in a humid chamber at 4°C. Secondary goat antibodies to mouse were Cy3 labeled (KPL, Guildford, UK) and diluted in 1:200 ratio. Slides were incubated with secondary antibodies for 40 minutes at room temperature. Chromosomes were counterstained with YOYO-1 (Invitrogen, Way Carlsbad, CA 92008 USA) and mounted in DABCO antifade solution (0.233 g DABCO, 800 μl H_2_O, 200 μl 1 M trisHCl pH 8.0, 9 ml glycerol). Slides were examined using a Zeiss LSM 510 Laser Scanning Microscope (Carl Zeiss MicroImaging Inc., Thornwood, NY, USA).

### GO annotation of heterochromatin and unmapped genome assembly

The *An. gambiae *AgamP4 annotated peptide set was analyzed using a locally installed copy of Interproscan 4.4.1 [84]. A GO [75] annotation file was generated using Interproscan-assigned GO terms and custom Perl scripts. Go-Term-Finder [85] version 0.86 was used to search for significantly overrepresented (i.e., p < 0.05) GO terms assigned to genes in heterochromatin relative to frequencies for all GO-annotated genes in the peptide dataset. All scores reported have been Bonferroni corrected to account for multiple comparisons. Genes within the euchromatin--heterochromatin transition zones were considered euchromatic for this analysis. Bar graphs were generated with Microsoft Excel and labeled using Adobe Illustrator CS4.

### Gene and repetitive element databases

Counts and length of coverage of all molecular features were identified in 5-Mb intervals in euchromatin and < 1-Mb intervals in heterochromatin of the *An. gambiae *AgamP3 genome assembly [76]. Gene density and TE coverage were analyzed using the Biomart [86] and RepeatMasker [87] (http://www.repeatmasker.org/) programs, respectively. Micro- and minisatellites were analyzed by Tandem Repeats Finder [88]. Only tandem repeats with 80% matches and a copy number of 2 or more (8 or more for microsatellites) were included in the analysis. Microsatellites, minisatellites, and satellites had period sizes ranging from 2 to 6, from 7 to 99, and from 100 or more, respectively. SDs were detected using BLAST-based whole-genome assembly comparison [89] limited to putative SDs represented by pair-wise alignments with ≥2.5-kb and >90% sequence identity. The alignment length was specifically chosen to avoid the vast majority of incompletely masked repetitive elements. SD counts are not discrete duplication events, but indicate the number of regions that have been involved in duplications within a given interval. Putative MARs in the *An. gambiae *genome sequence were predicted using the SMARTest bioinformatic tool [90].

### Bayesian statistical analysis of molecular features in the chromatin types

We have developed a model and procedure for discerning differences in molecular features between chromatin types. For this analysis, we incorporated data which distinguishes both the counts for each molecular feature and the overall coverage of each feature in subdivided regions of each of the five chromatin types of interest *ξ*_*i *_∈ *A *= {EU, PH, IHc, PEU, IHd}, where PH--pericentric heterochromatin, IHc--compact intercalary heterochromatin, IHd--diffuse intercalary heterochromatin, PEU--proximal euchromatin, EU--euchromatin. Since each region of the genome where these chromatin types are located is closely independent of each other, the likelihood follows as:

$$\prod_{\xi_{j}\in A}\prod_{i\in \xi_{j}}\mathrm{Pr}(C_{i,\xi_{j}}|\text{Data},\Theta ),$$

where $C_{i,\xi_{j}}$ are the counts associated with arm *ξ*_*j *_for chromatin type *i *and Θ are the unknown model parameters that must be estimated.

For our application, we used a Poisson random effects model for explaining the counts, but included information about the coverage in each region as well. To make this connection, we parameterized the mean effect $\lambda_{i,\xi_{j}}$ through the log-link function as:

$$\log(\lambda_{i,\xi_{j}})=\mu_{\xi_{i}}+\beta_{\xi_{i}}\log(L_{i})+\zeta_{\xi_{i}}\log(K_{i})$$

where *L*_*i *_is the total length and *K*_*i *_is the coverage length for chromatin type *i. *For each chromatin type, $\beta_{\xi_{i}}$ and $\zeta_{\xi_{i}}$ are random effects relating to the effect each length has on distinguishing the number of the molecular feature. $\mu_{\xi_{i}}$ relates to the overall density of the counts for each chromatin region. Hence in our case, the model unknowns are $\Theta =\{\mu_{\xi_{i}},\beta_{\xi_{i}},\zeta_{\xi_{i}}\}$ for each *ξ*_*i *_∈ *A*={EU, PH, IH c, PEU, IH d}.

Our ultimate goal was to determine if random effects $\left\{\mu_{\xi_{i}},\beta_{\xi_{i}},\zeta_{\xi_{i}}\right\}$ can be statistically distinguished between chromatin types. Dominant model selection procedures have the ability to compare all possible competing models and also to compensate for the number of parameters involved in each model. That is, if model fit is the objective, then all procedures will determine optimality by utilizing as many parameters as is possible. In our case, these could correspond to 125 possible parameter configurations. Since models selected this way are generally suboptimal in terms of prediction, likelihood penalization schemes are common practice. For instance, the Bayesian information criterion (BIC) and the Akaike information criterion (AIC) are commonly used devices for selecting between models. We relied on the former, since this criterion closely aligns with Bayes Factor computation. Explicitly, BIC, under model *M*_*k*_, is computed as:

$$-BIC_{M_{k}}=2\log(p(\text{Data}|\overset{\wedge }{M}))-\log(N)p$$

where $p(\text{Data}|\overset{\wedge }{M})$ is the maximum likelihood estimate (MLE), under model *k*, *N *is the number of observations, and *p *is the number of parameters in model *k*.

Bayes factors select between models through the ratio

$$BF=\frac{\int p(\text{Data}|\Theta ,M_{k})p(\Theta |M_{k})d\Theta }{\int p(\text{Data}|\Theta ,M_{\tilde{k}})p(\Theta |M_{\tilde{k}})d\Theta }$$

which can be interpreted as the level of support model *M*_*k *_has in favor of the data over model $M_{\tilde{k}}$. As an approximation, we have

$$e^{-\frac{1}{2}\Delta BIC}=e^{-\frac{1}{2}(BIC_{M_{k}}-BIC_{M_{\tilde{k}}})}\approx BF$$

which is a measure that decides between models and accounts for high degrees of observational variation. In order to compute the MLEs used in BIC calculations, we relied on an annealing algorithm. Specifically, given multiple locations in model space, state values in Θ and model configurations are simultaneously maximized to provide the MLE estimates for each data set. This procedure was repeated 1,000,000 times to ensure global optimization was achieved and that the best models (MAX models) were selected. The MAX models for each feature are given below.

RNA TEs: MAX model is (PEU = EU)--BIC = -2471.87, (PEU = EU, IHc = IHd)--BIC = -2474.12, and all different--(-BIC = -2475.64). So, PEU = EU has strong support (MAX model) over the model with all distinguishing chromatin types ΔBIC = 3.77, and ΔBIC = 1.52 for distinguishing models with all distinct from (PEU = EU, IHc = IHd), which is moderate support that euchromatin and intercalary heterochromatin types can be considered the same for retroelements. All other models have negligible support (ΔBIC > 10).

DNA TEs: MAX model is (IHc = PEU)--BIC = -1032.5, (IHc = PEU, IHd = PH)--BIC = -1034.0, so there is support for (IHc = PEU, IHd = PH) ΔBIC = 1.5. All other hypotheses ΔBIC > 9.

SDs: MAX model is (IHc = IHd)--BIC = -1540.2, all other hypotheses have ΔBIC > 8.

MARs: MAX model is (PH = IHc)--BIC = -1305.21, (PH = IHc = IHd)--BIC = -1306.44, (PH = IHc = IHd = PEU)--BIC = -1309.39. So, distinguishing IHd from (PH = IHc) has support ΔBIC = 1.23, which is mild. PEU is sufficiently different from each of the other candidate hypotheses, so we deem (PH = IHc = IHd). Differentiation from the all distinguishable model has ΔBIC > 10.

Genes: MAX model is (EU = PEU, PH = IH c = IH d)--BIC 468.96, ΔBIC > 10 for all nonnested hypotheses.

Microsatellites: MAX model is (IHc = PEU)--BIC = -1408.22, (IHc = PEU = IHd)--BIC = -1407.98, ΔBIC = 1.76. Supported hypothesis is (IHc = PEU = IHd).

Minisatellites: MAX model is (PEU = IHc)--BIC = -1887.03, (EU = PEU = IHc)--BIC = -1890.15 ΔBIC = 3.12, so supported hypothesis is EU = PEU = IHc, and less parsimoniously PEU = IHc. All other hypotheses have ΔBIC > 10.

Satellites: MAX model is (IHc = PEU)--BIC = -656.78, all other hypotheses have ΔBIC > 10.

## List of abbreviations

AIC: Akaike information criterion; BIC: Bayesian information criterion; BSA: bovine serum albumin; DABCO: 1,4-diazabicyclo[2.2.2]octane; EU: euchromatin; FISH: fluorescent *in situ *hybridization; GO: gene ontology; HP1: heterochromatin protein 1; IHc: compact intercalary heterochromatin; IHd: diffuse intercalary heterochromatin; *lhr*: *lethal hybrid rescue*; MAR: matrix attachment region; MLE: maximum likelihood estimate; MR4: Malaria Research and Reference Reagent Resource Center; NE: nuclear envelope; PBS: phosphate buffered saline; PH: pericentric heterochromatin; PEU: proximal euchromatin; SD: segmental duplication; SSC: saline-sodium citrate; TE: transposable element.

## Authors' contributions

IVS designed research; MVS, PG, IVB, SCL, JAB, CDS, and IVS performed research; SCL and JAB contributed new reagents/analytic tools; MVS, PG, IVB, SCL, JAB, CDS, and IVS analyzed data; and MVS, IVS and SCL wrote the paper. All authors read and approved the final manuscript.

## Supplementary Material

Additional file 1 **Table S1**. Coverage (%) of molecular elements in chromatin types of *An. gambiae*. Click here for file
