# Supplementary material for: Genome mapping and characterization of the Anopheles gambiae heterochromatin
Source: BMC Genomics. 2010 Aug 4;11:459. doi: 10.1186/1471-2164-11-459 (PMC3091655; doi:10.1186/1471-2164-11-459)
Supplement: Additional file 1 — Table S1. Coverage (%) of molecular elements in chromatin types of An. gambiae. [file 1471-2164-11-459-S1.DOC]

**Table S1. Coverage (%) of molecular elements in chromatin types of *An. gambiae*.**

| DNA transposons | | | | | |
| --- | --- | --- | --- | --- | --- |
|  | Euchromatin | Pericentric heterochromatin | Compact intercalary heterochromatin | Proximal euchromatin | Diffuse intercalary heterochromatin |
| Mean coverage | 1.558 | 9.374 | 3.235 | 4.297 | 9.720 |
| Median coverage | 1.340 | 9.325 | 3.100 | 3.100 | 10.840 |
| Mean ranks | 1.269 | 4.277 | 2.162 | 2.728 | 4.564 |
| RNA transposons | | | | | |
|  | Euchromatin | Pericentric heterochromatin | Compact intercalary heterochromatin | Proximal euchromatin | Diffuse intercalary heterochromatin |
| Mean coverage | 3.269 | 23.774 | 11.975 | 6.488 | 17.628 |
| Median coverage | 2.400 | 22.185 | 13.430 | 4.895 | 17.325 |
| Mean ranks | 1.194 | 4.766 | 3.102 | 2.103 | 3.835 |
| Segmental duplications | | | | | |
|  | Euchromatin | Pericentric heterochromatin | Compact intercalary heterochromatin | Proximal euchromatin | Diffuse intercalary heterochromatin |
| Mean coverage | 6.095 | 9.837 | 12.332 | 7.795 | 14.930 |
| Median coverage | 4.870 | 9.214 | 11.575 | 7.935 | 14.775 |
| Mean ranks | 2.149 | 3.196 | 3.274 | 2.611 | 3.770 |
| Microsatellites | | | | | |
|  | Euchromatin | Pericentric heterochromatin | Compact intercalary heterochromatin | Proximal euchromatin | Diffuse intercalary heterochromatin |
| Mean coverage | 0.441 | 0.693 | 0.452 | 0.433 | 0.370 |
| Median coverage | 0.370 | 0.685 | 0.410 | 0.465 | 0.330 |
| Mean ranks | 2.453 | 4.560 | 3.149 | 2.727 | 2.111 |
| Minisatellites | | | | | |
|  | Euchromatin | Pericentric heterochromatin | Compact intercalary heterochromatin | Proximal euchromatin | Diffuse intercalary heterochromatin |
| Mean coverage | 1.022 | 1.687 | 0.912 | 0.918 | 1.400 |
| Median coverage | 0.720 | 1.715 | 0.870 | 0.830 | 1.370 |
| Mean ranks | 2.397 | 4.259 | 2.400 | 2.212 | 3.732 |
| Satellites | | | | | |
|  | Euchromatin | Pericentric heterochromatin | Compact intercalary heterochromatin | Proximal euchromatin | Diffuse intercalary heterochromatin |
| Mean coverage | 0.296 | 0.573 | 0.088 | 0.147 | 0.405 |
| Median coverage | 0.230 | 0.580 | 0.090 | 0.075 | 0.200 |
| Mean ranks | 3.175 | 4.548 | 1.956 | 2.185 | 3.136 |
| MARs | | | | | |
|  | Euchromatin | Pericentric heterochromatin | Compact intercalary heterochromatin | Proximal euchromatin | Diffuse intercalary heterochromatin |
| Mean coverage | 3.393 | 6.373 | 5.850 | 7.487 | 6.320 |
| Median coverage | 3.400 | 5.895 | 5.900 | 7.075 | 6.540 |
| Mean ranks | 1.508 | 3.119 | 2.788 | 4.165 | 3.420 |
| Genes | | | | | |
|  | Euchromatin | Pericentric heterochromatin | Compact intercalary heterochromatin | Proximal euchromatin | Diffuse intercalary heterochromatin |
| Mean coverage | 5.684 | 1.440 | 1.287 | 6.400 | 1.455 |
| Median coverage | 5.780 | 1.400 | 0.820 | 6.230 | 1.580 |
| Mean ranks | 4.306 | 2.230 | 1.734 | 4.689 | 2.041 |
